# Secondary Care Clinic for Chronic Disease: Protocol

**DOI:** 10.2196/resprot.3902

**Published:** 2015-02-16

**Authors:** Clémence Dallaire, Michèle St-Pierre, Lucille Juneau, Samuel Legault-Mercier, Elizabeth Bernardino

**Affiliations:** ^1^Faculty of NursingUniversité LavalQuébec, QCCanada; ^2^Department of ManagementFaculty of AdministrationUniversité LavalQuébec, QCCanada; ^3^Centre d’Excellence sur le Vieillissement de QuébecHôpital du Saint-SacrementCHU de QuébecQuébec, QCCanada; ^4^Université LavalQuébec, QCCanada; ^5^Department of NursingFederal University of ParanáCuritibaBrazil

**Keywords:** delivery of health care, integrated, chronic disease, secondary care, tertiary health care, ambulatory care, research design, models, theoretical

## Abstract

**Background:**

The complexity of chronic disease management activities and the associated financial burden have prompted the development of organizational models, based on the integration of care and services, which rely on primary care services. However, since the institutions providing these services are continually undergoing reorganization, the Centre hospitalier affilié universitaire de Québec wanted to innovate by adapting the Chronic Care Model to create a clinic for the integrated follow-up of chronic disease that relies on hospital-based specialty care.

**Objective:**

The aim of the study is to follow the project in order to contribute to knowledge about the way in which professional and management practices are organized to ensure better care coordination and the successful integration of the various follow-ups implemented.

**Methods:**

The research strategy adopted is based on the longitudinal comparative case study with embedded units of analysis. The case study uses a mixed research method.

**Results:**

We are currently in the analysis phase of the project. The results will be available in 2015.

**Conclusions:**

The project’s originality lies in its consideration of the macro, meso, and micro contexts structuring the creation of the clinic in order to ensure the integration process is successful and to allow a theoretical generalization of the reorganization of practices to be developed.

## Introduction

### Context

In Canada and around the world, the growing complexity and financial burden of chronic disease prevention and management are increasingly prompting the development of organizational models based on interdisciplinarity and integrated care networks. Most of these models were meant to be implemented at the primary health care services organization level [1-6]. However, during recent years in the province of Quebec, the primary care level has been impacted by many changes, including institutional mergers, service pathway redesign, and increased responsibilities. All these changes have still not yet been fully absorbed today [7]. In this context and following on from the “National Consultation on Health Services and Policy Issues 2007-2010” [8], which suggests that innovative models should take particular geographic contexts and existing resources into account, the Centre hospitalier affilié universitaire de Québec, a specialty and subspecialty care facility, plans to open a clinic dedicated to chronic disease. The Centre will work with the existing clinics at the two hospitals that treat diabetes, heart failure, chronic obstructive pulmonary disease, and inflammatory bowel disease to improve care organization and management and, ultimately, ensure greater coordination and integration of the different follow-ups required for each of these diseases. The latter are generally recognized as priorities by the Public Health Agency of Canada [9], Quebec’s Ministère de la santé et des services sociaux [10,11], and the Regional Health Agency [12].

The Centre’s objectives are to reduce complications in the treatment of chronic disease, improve the quality of life of the chronically ill, create a pathway that will help alleviate overcrowding in emergency departments, and reduce the costs of treating these diseases. In the context of a hierarchy of care and services, the clinic set up by the Centre’s two hospitals (secondary and tertiary care) will have linkages to primary care services. Given the systemic challenges involved in creating the clinic, the Centre’s directors, through its Care and Services Coordination Committee, called on the expertise of researchers from Université Laval. The challenge is to reorganize activities based on current knowledge of the chronic care organization while also ensuring the initiative is a success by taking into account the imperatives underpinning the existing professional and management practices concerned. A preliminary study was therefore conducted by the research team, which obtained a training award in summer 2010 that was used to do a literature review as well as a grant from the Canadian Institutes of Health Research in April 2011 that was used to determine the components of the project to be implemented, identify the initial clinics and follow-ups to be set up with all the organizational and professional partners involved in the project, and prepare an overview of the situation.

### Chronic Diseases

Chronic diseases are pathologies that are often characterized by a long period of latency and a prolonged course causing functional impairment and multiple disabilities [13]. While incurable, these diseases are distinctive in that their symptoms and progression can be controlled, in particular through prevention, healthy lifestyle choices, and appropriate medical follow-up [7,11,14,15].

The leading cause of death worldwide [16], chronic diseases have devastating consequences both for patients and their families, but also for health care systems [14,17-19]. The decline in quality of life due to the inability to perform daily activities, absenteeism, productivity losses [18,20-22], as well as the personal, emotional, and social burden for those affected are among the costs associated with these diseases. On a structural level, the cost of disability, morbidity, and death due to chronic disease in Canada is over CAN $80 billion [22]. For instance, patients with chronic diseases are among the most frequent users of emergency departments and are also known for their recurrent hospitalizations [23,24]. Given these findings, similar to various international bodies, the Canadian Health Council acknowledges that current approaches to chronic disease organization and management are contributing to ever-increasing costs and wait times [18,19,23]. Within Canada and internationally, care pathways are inadequate and access to care and services is difficult [7,18,25-27]. These problems are attributed to the fact that the current culture and structure of health care services delivery are reactive, since they are designed to systematically relieve symptoms and treat acute episodes [1,17,25,28-37]. Consequently, many authors [1,2,4,7,10,37-39] believe health care transformation must be achieved by introducing a system that adopts an integrated prevention and management strategy where patients are followed long term and play an active role in their health, and that is based on a systematized, proactive, and coordinated approach.

### Chronic Care Management Models

Many countries have recently proposed various effective projects [17,33,40-48] for chronic disease management that are based on care management [49]. Improved health outcomes can be achieved by reducing the risks of complications, while people’s functional abilities can be maintained and their professional care requirements reduced by more clearly identifying the care and services needed [6,7,25,27,28]. To achieve this, management must be coherent and coordinated in order to provide assistance and therapeutic management for the person in the long term [4,7,10,19,37,38]. While the emphasis is at times placed on early screening and adapted management (Kaiser Permanente, Guided Care, EverCare, Pfizer) [7,27,29,30,50,51], at times on making patients take more responsibility for their health combined with case management by specialized nurses rather than medical specialists (Community Matrons, Social Care Model) [7,51], and at times on multidisciplinary work (Veterans Health Administration) [5,38,51], the Chronic Care Model [36] remains the most comprehensive and widely used of these integrated management models [8,24,28,38,47].

Taking a holistic approach, the Chronic Care Model [52] encompasses six areas for concerted action: (1) delivery system redesign, (2) support for patient self-management, (3) clinical information systems, (4) clinical decision support, (5) community partnerships, and (6) health care organization and leadership. It is based on a clearly established partnership between informed, proactive people living with chronic conditions and trained, supported health care teams [6,17,25,28,35,51,53]. Numerous studies have documented the positive effects of implementing the six components of the Chronic Care Model, including reduced costs and improved client satisfaction, quality, and clinical outcomes [39,51,54]. However, since a number of barriers related to professional practices and management methods appear to prevent the desired approach from being established, Sunaert et al [47] point out that it is crucial to highlight the complementary and unique nature of the contributions and tasks of each of the stakeholders involved in the care process. To do so, they suggest implementing the components of the model progressively to allow stakeholders to fully absorb the changes. These findings have also been confirmed by various studies that suggest that it is not necessary to implement all the components of the Chronic Care Model at once for it to be effective [6,7,17,38,39,51,52].

### The Centre Hospitalier Affilié Universitaire de Québec Project

At the Centre, therefore, the decision will be to implement those components that would be most likely to ensure the initiative is a success by harmonizing the different practices in the participating hospitals, beginning with two specific clinics.

The components were selected based on Si and Bailie’s study [55] that, following a review of 69 projects featuring elements of the Chronic Care Model, identified the four components an organization can focus on to ensure stakeholders make the model their own and implement the underlying elements. These components are delivery system redesign, support for patient self-management, clinical information systems, and clinical decision support.

Delivery system redesign involves defining the roles of care providers in the division of tasks [28,32,35,44,56,57], creating interdisciplinary teams, as well as coordinating patient follow-up and access to services [56,58]. In this respect, the Centre plans to clarify the roles of professionals—physicians, nurses, nutritionists, pharmacists, psychologists—in the assessment of the patient’s initial condition, patient education, and types of follow-up. Activities already set up will be consolidated by adding specialized nurses, establishing a point-of-access for clients, and possibly by introducing walk-in access to medical specialists. At the Centre, the stakeholder also plans to develop linkages with family physicians who provide primary care by systematically informing them of the patient’s condition in a manner that is acceptable to all.

Self-management support involves encouraging patients to make behavior and lifestyle changes [35,38,44,56,57] while providing them with the tools they need to perform the necessary monitoring and to seek care in specific situations [6,15,17,28,55,57,59]. For the Centre, this means strengthening support activities for patients, organizing support activities for the patients’ families at group meetings, and developing a follow-up plan that will be reviewed periodically.

Clinical decision support involves offering continuing education activities to providers, developing various consultation mechanisms for specialists, ensuring easy and rapid access to specialist expertise, as well as producing evidence-based interdisciplinary practice guidelines and protocols [7,28,34,35,38,44,47]. At the Centre, bringing various professionals from the two hospitals under the same roof in the chronic disease clinic and promoting evidence-based practices are the two prerequisites needed to develop support activities.

Clinical information systems development involves creating a system accessible to professionals that contains clinical information about patients and the various parameters monitored and required to adjust care [6,15,17,28,35,55,57,60]. To this end, driven by the concerns of physicians, a patient registry could be created at the Centre hospitalier affilié universitaire de Québec so that all care providers can rapidly input and share basic clinical data on the patient’s condition, enter reminders for periodic follow-ups, and access certain patient data. The introduction of patient assessment and follow-up tools is also being considered by the physicians to facilitate intra- and interdisciplinary work.

To ensure the feasibility of the project, existing clinical and organizational realities took precedence when selecting the clinics and conditions to be followed. Thus, diabetes and inflammatory bowel disease will lay the groundwork for the Centre hospitalier affilié universitaire de Québec’s chronic disease clinic. The selection of diabetes came from the Centre’s directors, while inflammatory bowel disease came from the professionals themselves. In the case of diabetes, the directors’ decision to restructure the two hospitals under their jurisdiction prevailed in order to strengthen each site’s mission, namely, to develop ambulatory care at a single site. This decision was made to better address the specific needs of clients by providing access to integrated ambulatory services rather than having clients use emergency services and thereby make more efficient use of professional resources, space, and technical platforms. In the case of inflammatory bowel disease, the physician who leads the department of gastroenterology, using funding obtained to provide the services of a specialized nurse, submitted a request to the Centre’s directors to introduce a more integrated follow-up. This would involve redesigning the pathway of patients who systematically go to the emergency department. In addition to improving patients’ quality of life and their satisfaction with care, the objective of this initiative is to bring down the costs of repeated hospitalizations, decrease the number of tests currently ordered by emergency physicians due to their inability to access records, and reduce the use of costly medications.

These two initial clinics that will be set up within the same hospital will provide complementary internal and external services (primary care services), while relying on the fact that the latter are currently integrating various services, including community services, long-term residential services, and certain ambulatory services. This study is therefore contributing to the development of the local services networks that have been created to promote collaboration between the various care professionals in a given territory [61].

### Conceptual Framework and Objectives

**Figure 1:**
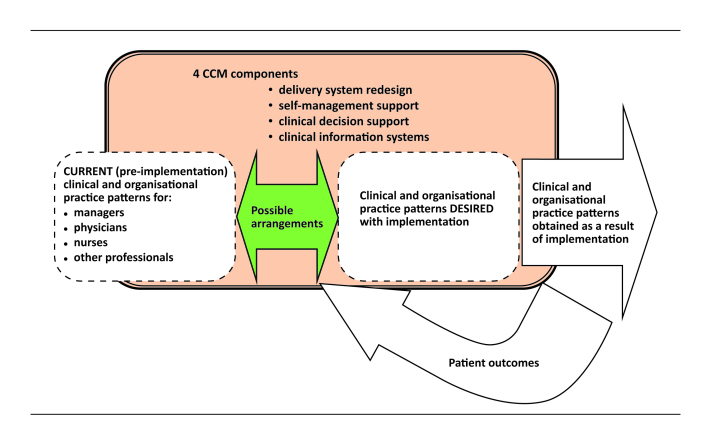
Framework of the chronic disease clinic.

Since the creation of the chronic disease clinics must be approached in a strategic manner, the research project is based on an analysis of practices in the different groups of stakeholders concerned (clinical and organizational practices of managers, medical specialists, nurses, and other professionals—pharmacists, psychologists, nutritionists—and primary care physicians). It involves analyzing existing dynamics and those that might ensue from the implementation of the four selected components of the Chronic Care Model within the continuum of services [62-65]. Figure 1 provides a summary of the framework that will be used in the research.

The general objectives of the research project are (1) to produce knowledge that will allow what is learned from the two diabetes and inflammatory bowel disease projects to be used in a complementary manner to set up a clinic at some point in the future that will treat a wider range of chronic diseases, and (2) to build on this knowledge to construct an explanatory theoretical model that will contribute to the development of similar programs or projects in other regions of Quebec, Canada, and other countries, including Brazil, the country of origin of one of the researchers involved in this research project. The specific objectives are shown in Textbox 1.

**Objectives of the research project.**

Objective 1. For each of the two clinics to be set up:

1.1 To describe and analyze current practice patterns and the arrangements that may be used by each of the groups of stakeholders involved in the activities underlying the four components of the Chronic Care Model.

1.2 To understand the clinical and organizational practices that result from these practice patterns and arrangements during implementation.

1.3 To assess patient outcomes for each of the diseases studied.

Objective 2. For both clinics:

To compare and contrast clinical and organizational practices for the diseases studied in order to assess their impact.

Objective 3. To develop a model to redesign the delivery of services to people with chronic diseases.

## Methods

### Research Strategy

The strategy adopted is based on the longitudinal comparative case study method with embedded units of analysis [66]. This strategy is especially relevant when studying contemporary events over which the researcher has little or no control. Moreover, a case study is the preferred strategy, since it allows the implementation of follow-ups to be studied in depth and in the real-life context of a university hospital center where multiple factors come into play when effecting clinical and organizational changes. More specifically, a case study will allow clinical and organizational practices related to the project to implement two chronic disease clinics and follow-ups to be detailed based on the expertise used to carry out activities (knowledge, know-how), the roles played (tasks and function), the powers deployed (influence, capacity for action), and supporting organizational methods [65]. Thus, the underlying conditions of the follow-ups and their impact on the implementation of the four components of the Chronic Care Model will be documented. This approach will allow us to study how the Chronic Care Model can be adapted to the clinical and organizational practices in a specific organization and how, from there, insight can be gained into the organization of chronic disease management.

To meet the specific objectives of the research project, namely, to analyze clinical and organizational practices while taking into account their impact on patients, the case study will include a mixed research method [67]. A descriptive, explanatory, and comprehension-oriented qualitative approach will be used to gain a better understanding of the dynamics at work in the redesigns in question (Objectives 1.1, 1.2, and 2) in order to produce a redesign model that takes these dynamics into account (Objective 3). Quantitative methodology will be used to complete the qualitative component in order to assess patient outcomes following implementation (Objective 1.3).

### Sources of Data

To achieve Objectives 1.1 and 1.2, data will be collected by consulting management documents, conducting individual and group interviews, and by observation.

The management documents consulted for the chronic diseases studied will include specific documents used in the management of each of the diseases studied (policies, practice guidelines, minutes of meetings) and administrative documents (planning statements, action plans, reports, minutes). This literature search will be performed, in particular, to put the general context in which the two chronic disease clinics will be created into perspective (linkages with other partners in the local network, decisions and negotiations with regional and central bodies, decisions made by the Centre, key individuals, and resources allocated).

Semi-structured individual and group interviews with members of work groups from the Centre associated with the reorganization of chronic disease management and other managers, physicians, and professionals directly involved in implementing the follow-ups will also be held. These interviews will be conducted at four points in time: (1) at the start of implementation, (2) during implementation (6 months later), (3) 1 year after implementation, and (4) 18 months after the start of implementation. These four points in time were selected to take into account a possible difference in the implementation or rate of implementation between the two clinics. The interviews will be conducted using a checklist that documents (1) stakeholders’ roles depending on the nature of the work to be carried out (initial patient assessment, diagnosis, treatment, teaching, and types of patient follow-ups), (2) the expertise required depending on the nature of activities, self-management of care, and interprofessional and interorganizational communications, (3) the powers deployed with respect to the coordination of care and services with and between professionals, and (4) organizational supports for delivery, self-management, education, and information system activities.

Last, non-participant observation will be used within the management committees involved in setting up each clinic. Activities implemented to provide the follow-up for the two chronic diseases (education sessions, interventions of professionals who provide follow-ups and those of managers involved in implementation) will also be observed. This data collection will complete the information collected in the interviews throughout implementation and will be used to prepare subsequent interviews. This data will also be used to document the dynamics between managers and professionals and between professionals with respect to the possible clinical and organizational arrangements.

It should be noted that the preliminary data collection, carried out using the grant obtained from the Canadian Institutes of Health Research in 2011, revealed a number of barriers to the implementation of follow-ups, some related to the resources to be mobilized and others to clinical practices, which provides starting points for more extensive data collection.

To achieve Objective 1.3, data collection will involve consulting patient registries and distributing standardized questionnaires to patients. This data collection will begin 1 year after implementation and will extend over an 18-month period to take into account the arrival of new patients and the planned length of follow-up for each patient. In cases where follow-up is planned for the person’s life span, data will be collected at two points in time: 1 year after the start of implementation and 18 months later in order to document the impact of the follow-up provided on the person’s condition.

In addition to demographic data, the information collected from registries will include the patient’s state of health (main diagnosis, comorbidities, medication), the professional(s) consulted, the diagnosis or reason for consulting, the type of intervention, the intensity of services provided (number of contacts, their duration, and the length of follow-up), referral to a specialist or other professional, and, where applicable, the reason for terminating follow-up or destination following discharge. The patient registry should also document the impact on patients. Certain information will be collected in this regard, including test results (eg, repeat blood glucose tests, weight).

The impact will also be assessed by distributing standardized questionnaires to patients. These assessments will be used to study physical, social, family, and emotional functioning and will be completed during follow-up activities. Since the questionnaires will be administered by professionals, they will be designed to take into account the manner in which they will be used to assess patients’ functional capacities depending on the follow-up provided. Particular attention will be paid to the quality of teaching and information provided to facilitate self-management of care, as well as the intention to terminate follow-up. To do this, standardized questionnaires that are used with hospitalized patients will be adapted, in particular for the scales that assess difficulties adapting following discharge from hospital and the quality of discharge teaching. The scale used to assess difficulties adapting is divided into four attributes used to assess readiness based on personal status, knowledge, ability to self-manage, and support expected. It is a self-administered questionnaire that uses a Likert scale of 0 to 10. The scale used to assess the quality of teaching is divided into two subscales: one for content and the other for nurses’ teaching skills. The content assessment evaluates perception of the content required, the content received, and the difference between the two. For nurses’ skills, sensitivity to personal beliefs and values, the use of understandable language, and the choice of the best time to give the patient and his/her family the teaching are evaluated. This questionnaire is also self-administered and uses a Likert scale of 0 to 10.

### Recruitment of Participants

The project was submitted to and approved by the ethics committee of the site where the study will be conducted. It was agreed that all participants would be recruited on a voluntary basis.

Professionals and managers will first be recruited from among the initial contacts made by the researchers when they participated in the various committees involved in setting up the two clinics. All the other stakeholders directly involved in setting up these clinics will then be contacted personally by a member of the research team to invite them to participate. Patients will be recruited by someone who is neither involved in clinical follow-ups, nor part of the research team. Once they have agreed to participate, one of the members of the research team will contact them to explain the study.

All participants will be required to give their consent to participate in the research project by signing a form that guarantees the right to withdraw at any time during the process without prejudice. For patient consent to the use of their data, the rules proposed by the Canadian Institutes of Health Research, the Natural Sciences and Engineering Research Council of Canada, and the Social Sciences and Humanities Research Council will be followed [68]. The confidentiality of the data collected will be safeguarded by creating a coding system to ensure participants’ anonymity. All confidential data will be kept under lock and key. In accordance with applicable retention rules, documents relating to the project will be kept for 7 years after completion of the study [69].

### Data Analysis

Data analysis will be organized in accordance with the above-mentioned research objectives and guided by the dimensions of the conceptual framework (see Figure 1), the current (pre-implementation) clinical and organizational practices, the desired practices based on the Chronic Care Model, the possible arrangements between the two, and the results obtained in terms of practices implemented and patient outcomes. The data studied will include interview transcripts, data from observation, and data from the literature review; the resulting set of data will be transferred onto a computerized platform and analyzed using qualitative analysis software specially designed for mixed methods research (QDA miner: Provalis Research).

The qualitative data for Objectives 1.1 and 1.2 as well as 2 and 3 will be analyzed using Miles and Huberman’s methods [70].

First, for Objectives 1.1 and 1.2, the data collected will undergo an initial classification, after time 1, according to the dimensions of the conceptual framework. The data will then be reduced by grouping meanings according to the themes that emerge. Matrices and diagrams will then be used to highlight the patterns in the data. This operation will serve to establish associations between the different constructs of interest in the clinical and organizational practice patterns. Subsequently, at times 2, 3, and 4, the data will contribute to the themes already constructed and possibly reveal other explanatory patterns.

Objectives 2 and 3 concern, respectively, cross-sectional analyses of the practices at each clinic studied and the development of a model to redesign the delivery of services to people living with chronic diseases. For Objective 2, the different patterns of clinical and organizational practices will be taken, merged, and compared in order to produce a synthesis. Last, the model that must be constructed to meet Objective 3 will require the use of what Nizet [71] calls the double hermeneutic. Thus, the interpretations of the two analyses must be compared with the interpretations of managers and professionals at the clinics in order to achieve a global understanding of the situation.

Objective 1.3 will be achieved by conducting a quantitative data analysis using descriptive statistical analyses such as the mean, standard deviation, minimum and maximum and the quartiles of length of follow-ups, the number of professionals involved, the type of intervention, and the intensity of interventions. The analyses will explore the distribution of these variables. In addition to the impact on patients, the quantitative analysis of this data will complete Objective 2 and thus allow the quantifiable aspects of the two types of follow-up to be compared and contrasted, in particular the number of professionals involved and the intensity of interventions, the duration, and the variety of the types of interventions used.

Last, the validity of the analytical approach used in the study will be strengthened by using a variety of sources of data and building a “chain of evidence” starting with this data and ending with the construction of the explanations concerning clinical and organizational practices associated with delivery system redesign, self-management support, clinical decision support, and information systems. These explanations will form the basis of a cross-sectional explanation for the construction of a more comprehensive redesign model.

## Results

We are currently in the analysis phase of the project. The results will be available in 2015.

## Discussion

### Contributions and Limitations

The contributions of the research will be threefold: the first is to consider specialized services as the linchpin for the creation of chronic disease clinic; the second is to adapt an organizational model for chronic disease management to existing resources and practices; while the third is to provide a theoretical explanation that goes beyond the local example.

In the literature, the implementation of a model for the follow-up of chronic diseases is based on primary health care services. However, according to studies by Lévesque et al [7,38], the latter are still not sufficiently organized in Quebec to support such an initiative alone. The significant changes these services have undergone in recent years (institutional mergers, service pathway redesign, the creation of local services networks, increased responsibilities) have not yet all been absorbed such that linkages can be made between the multiple partners (hospital centers, medical specialist groups, health and social services centers, medical clinics) required to set up these clinics. This organizational reality combined with the fact that chronic diseases are often followed by hospital-based medical specialists suggest that the desired complementarities can be sought outside primary care services. Another form of care organization must be considered, which can only increase the possibility of improving chronic disease management.

Similarly, beyond the idea of implementing an organizational model created elsewhere, it is a question of basing implementation on actual practices and existing resources so that these practices and resources can be taken into account and the organizational model adapted to the organizational reality. Thus, the opposite approach is taken: instead of practices adapting to the model, the model adapts to practices in order to ensure implementation is successful and to ensure the long-term sustainability of the clinic in the follow-up of chronic diseases. This approach leads us to identify explanatory elements by describing and highlighting the dynamics at work that can improve our understanding of approaches to implementation that retain existing elements, to use them, and to add elements modelled on the literature and research evidence. This method is consistent with approaches to change where the emphasis is shifted from managing change to managing the capacity to change [72].

Last, such empirical perspectives are necessarily reflected on a scientific level. This research project therefore aims to develop a new theory of care organization. Since the dimensions of the Chronic Care Model already form the basis of stakeholders’ existing practices (delivery system redesign, self-management support, clinical decision support, and clinical information systems), but little is said about how to build on pre-implementation practices when new organizational models are introduced, developing a framework for the approach seems relevant. The intention is to go beyond the local example, to develop a model at a level that will allow generalizations of a theoretical nature. Developing knowledge of how to implement an integrated model for chronic disease follow-up is therefore highly relevant for the Canadian health care system and other systems around the world, service providers, and the chronically ill. Carrying out a project that can be used to document the adaptations that must be made to an intervention model based on evidence can only help improve the chances of success when setting up chronic disease clinics and thereby ensure their sustainability.

The main limitation of this study is that only one case is considered. However, this is minimized by the nature of the study and the possibility of making a theoretical generalization. While the description of the implementation cannot be generalized to other situations, the development of cross-sectional proposals of rationalization of the phenomenon of care organization and management for the chronically ill could possibly be validated in other health system organizations.

### Application of Integrated Knowledge

Collaboration between managers/decision makers, researchers, and professionals has existed for several years in the context of other projects. From the earliest planning stages of the ambulatory clinic at the Centre hospitalier affilié universitaire de Québec, the managers called on the expertise and support of researchers, for they wanted to reorganize follow-up activities for chronic diseases based on current knowledge. They also wanted to ensure the initiative was a success by taking professional practices and their specific context into account.

Thus, the knowledge application process is integrated into the research itself and consists of mutual exchanges throughout the study. The researchers participate in the Care and Services Coordination Committee, and the various other committees formed with professionals and managers involved in implementing the two follow-ups. The participation of a Brazilian researcher will contribute a point of view that will enrich local analysis perspectives, since services integration is an integral part of Brazil’s health system. To this end, discussions will also be held by videoconference with a consultation committee made up of decision makers and researchers associated with this researcher’s university and thereby contribute to the transfer of knowledge.

A workshop will be organized to which experts, decision makers, and researchers will be invited in order to discuss the findings of the study and the resultant service delivery redesign model. Moreover, various means of transferring knowledge, such as scientific and popular communications and publications, will be used to reach the research project’s target audience. Dissemination of the results in decision making, administrative, and professional circles will be planned, taking their specific characteristics into account. The results will be documented in the form of short notes, brief reports, and summaries. This information will be available at conferences attended by managers (Association des gestionnaires des établissements de santé et de services sociaux, Association des cadres de la santé et des services sociaux du Québec, Association des Directeurs généraux des Services de Santé et Sociaux du Québec).

## Multimedia Appendix 1

CIHR decision letter.

## Multimedia Appendix 2

CIHR ranking.

## Multimedia Appendix 3

CIHR peer-reviewer 1.

## Multimedia Appendix 4

CIHR peer-reviewer 2.
