# Supplementary material for: Secondary Care Clinic for Chronic Disease: Protocol
Source: JMIR Res Protoc. 2015 Feb 16;4(1):e12. doi: 10.2196/resprot.3902 (PMC4376234; doi:10.2196/resprot.3902)
Supplement: Supplementary file 1 [file resprot_v4i1e12_app1.pdf]

CIHR IRSC

Institute of Aboriginal Peoples' Health
Institute of Aging
Institute of Cancer Research
Institute of Circulatory and Respiratory Health
Institute of Gender and Health
Institute of Genetics
Institute of Health Services and Policy Research
Institute of Human Development and Child and Youth Health
Institute of Infection and Immunity
Institute of Musculoskeletal Health and Arthritis
Institute of Neurosciences, Mental Health and Addiction
Institute of Nutrition, Metabolism and Diabetes
Institute of Population and Public Health

Institut de la santé des Autochtones
Institut du vieillissement
Institut du cancer
Institut de la santé circulatoire et respiratoire
Institut de la santé des femmes et des hommes
Institut de génétique
Institut des services et des politiques de la santé
Institut du développement et de la santé des enfants et des adolescents
Institut des maladies infectieuses et immunitaires
Institut de l'appareil locomoteur et de l'arthrite
Institut des neurosciences, de la santé mentale et des toxicomanies
Institut de la nutrition, du métabolisme et du diabète
Institut de la santé publique et des populations

Le 30 avril 2012

Docteure Clémence DALLAIRE
Faculté des sciences infirmières
1050, rue de la Médecine
Pavillon Ferdinand Vandry
Université Laval
Québec, Québec G1V 0A6

OBJET : Subvention Partenariats pour l'amélioration des services de santé : Concours 2011-2012, « L'implantation d'une clinique de suivis intégrés de maladies chroniques en misant sur la deuxieme ligne de soins ».

Docteure DALLAIRE,

Au nom de la Direction de l'application des connaissances et de ses partenaires, nous sommes heureux de vous annoncer que la demande susmentionnée a été retenue pour du financement. Vous recevrez votre autorisation de financement sous peu par la poste.

Le programme « Partenariats pour l'amélioration des services de santé » est dirigé par la Direction de l'application des connaissances. Veuillez noter que votre subvention est financée par la Direction de l'application des connaissances des IRSC et l'Initiative « Renouvellement des soins de santé fondé sur des données probantes » des IRSC. Vous êtes tenus de faire mention de l'aide des IRSC et des partenaires financiers pertinents dans les communications et publications relatives à votre projet.

La subvention des IRSC demeure conditionnelle à la contribution de vos partenaires tel que décrit dans votre demande de subvention. Veuillez consulter l'annexe en pièce jointe pour connaître l'engagement de vos partenaires de concours et de projet. Veuillez aviser les IRSC s'il y a une diminution de l'engagement de l'un de vos partenaires.

Étant donné que les IRSC n'informent plus les co-candidats de leur décision, nous vous prions de communiquer le résultat de cette demande aux personnes concernées et à leur établissement de recherche (s'il diffère du vôtre).

Un rapport final devra obligatoirement être présenté aux IRSC. Les IRSC fourniront un modèle normalisé de ce rapport et vous aviseront de la date limite pour le soumettre.

Pour de plus amples renseignements sur le processus d'examen, veuillez communiquer avec Anne-Marie Poulin, coordonnatrice, Exécution des programmes, par courriel à anne-marie.poulin@cihr-irsc.gc.ca ou par téléphone au 613-948-2899.

Nous vous souhaitons un franc succès dans vos travaux de recherche.

Veuillez agréer, Docteure DALLAIRE, l'expression de mes meilleurs sentiments.

Alison Bourgon
Directrice adjointe intérimaire, Direction des programmes de création de connaissances
Portefeuille de la recherche

c.c. Agent financier, Institution payée
Administrateur de recherches, Institution payée

298966-201111PHE-PHE-267464-94456-PHEA

Canadian Institutes of Health Research
Room 97, 160 Elgin Steet, Address locator: 4809A
Ottawa, (Ontario) K1A 0W9 Tel.: (613) 941-2672
Fax (613) 954-1800 **www.cihr-irsc.gc.ca**

Instituts de recherche en santé du Canada
Pièce 97, 160 rue Elgin, Indice de l'adresse: 4809A
Ottawa, (Ontario) K1A 0W9 Tél.: (613) 941-2672
Fax (613) 954-1800 **www.irsc-cihr.gc.ca**

Canada
