# Supplementary material for: Secondary Care Clinic for Chronic Disease: Protocol
Source: JMIR Res Protoc. 2015 Feb 16;4(1):e12. doi: 10.2196/resprot.3902 (PMC4376234; doi:10.2196/resprot.3902)
Supplement: Supplementary file 2 [file resprot_v4i1e12_app2.pdf]

April/Avril 30, 2012

DC0190GP

Canadian Institutes of Health Research / Instituts de recherche en santé du Canada

Notice of Decision / Avis de décision

Application Number/Numéro de la demande: 267464

Committee Code/Code du comité: PHE

Applicants/Candidats: Docteure Clémence DALLAIRE   Madame Lucille JUNEAU

With/Avec: Mademoiselle K. AUBIN   Docteur E. BERNARDINO   Docteur C. GAUTHIER   Mademoiselle M. ST-PIERRE

Institution paid/ Établissement payé: Université Laval

Title/Titre: L'implantation d'une clinique de suivis intégrés de maladies chroniques en misant sur la deuxieme ligne de soins

Primary Inst./ Inst. principal: Services et politiques de la santé

Other Related Inst./ Autres inst. connexes:

Competition Outcome/Résultats du concours: Partenariats pour l'amélioration du système de santé (PASS)

November/Novembre 01, 2011

Number in competition/Nbre de demandes dans le concours: 61

Number approved/Nbre de demandes approuvées: 27

Decision on your application/ Décision sur votre demande: Approuvée

Average annual amount/ Montant annuel moyen: $97,811

Equipment amount/ Montant pour les appareils: $0

Term/Durée: 3 **yrs/ans** 0 **months/mois**

Peer Review Committee Recommendation, for your information and use/ Recommandation du comité d'examen par les pairs, pour fins d'information et d'utilisation:

Committee/Comité: Partenariats pour l'amélioration du système de santé

Application rank within the competition/ Rang de la demande dans ce concours: 21

Percent Rank Within the Competition/ Rang en pourcentage au sein du concours: 34.43%

Rating/ Cote:

Répercussions possibles	4.01
Mérite scientifique	3.66

Recommended average annual amount/ Montant annuel moyen recommandé: $97,811

Recommended equipment amount/ Montant recommandé pour les appareils: $0

*** Applications receiving a score of less than 3.5 on any evaluation criteria will not be considered for Funding. / Les demandes qui ont reçu une note inférieure à 3.5 pour n'importe quel des critères d'évaluation ne sont pas admissibles.

This document is for information only. Official payment is stated on the CIHR Authorization for Funding.
Document à titre d'information seulement. Le paiement officiel est indiqué sur l'autorisation de financement des IRSC.

CIHR IRSC

Institute of Aboriginal Peoples' Health
Institute of Aging
Institute of Cancer Research
Institute of Circulatory and Respiratory Health
Institute of Gender and Health
Institute of Genetics
Institute of Health Services and Policy Research
Institute of Human Development and Child and Youth Health
Institute of Infection and Immunity
Institute of Musculoskeletal Health and Arthritis
Institute of Neurosciences, Mental Health and Addiction
Institute of Nutrition, Metabolism and Diabetes
Institute of Population and Public Health

Institut de la santé des Autochtones
Institut du vieillissement
Institut du cancer
Institut de la santé circulatoire et respiratoire
Institut de la santé des femmes et des hommes
Institut de génétique
Institut des services et des politiques de la santé
Institut du développement et de la santé des enfants et des adolescents
Institut des maladies infectieuses et immunitaires
Institut de l'appareil locomoteur et de l'arthrite
Institut des neurosciences, de la santé mentale et des toxicomanies
Institut de la nutrition, du métabolisme et du diabète
Institut de la santé publique et des populations

Le 30 avril 2012

Docteure Clémence DALLAIRE
Faculté des sciences infirmières
1050, rue de la Médecine
Pavillon Ferdinand Vandry
Université Laval
Québec, Québec G1V 0A6

OBJET : Subvention Partenariats pour l'amélioration des services de santé : Concours 2011-2012, « L'implantation d'une clinique de suivis intégrés de maladies chroniques en misant sur la deuxieme ligne de soins ».

Docteure DALLAIRE,

Au nom de la Direction de l'application des connaissances et de ses partenaires, nous sommes heureux de vous annoncer que la demande susmentionnée a été retenue pour du financement. Vous recevrez votre autorisation de financement sous peu par la poste.

Le programme « Partenariats pour l'amélioration des services de santé » est dirigé par la Direction de l'application des connaissances. Veuillez noter que votre subvention est financée par la Direction de l'application des connaissances des IRSC et l'Initiative « Renouvellement des soins de santé fondé sur des données probantes » des IRSC. Vous êtes tenus de faire mention de l'aide des IRSC et des partenaires financiers pertinents dans les communications et publications relatives à votre projet.

La subvention des IRSC demeure conditionnelle à la contribution de vos partenaires tel que décrit dans votre demande de subvention. Veuillez consulter l'annexe en pièce jointe pour connaître l'engagement de vos partenaires de concours et de projet. Veuillez aviser les IRSC s'il y a une diminution de l'engagement de l'un de vos partenaires.

Étant donné que les IRSC n'informent plus les co-candidats de leur décision, nous vous prions de communiquer le résultat de cette demande aux personnes concernées et à leur établissement de recherche (s'il diffère du vôtre).

Un rapport final devra obligatoirement être présenté aux IRSC. Les IRSC fourniront un modèle normalisé de ce rapport et vous aviseront de la date limite pour le soumettre.

Pour de plus amples renseignements sur le processus d'examen, veuillez communiquer avec Anne-Marie Poulin, coordonnatrice, Exécution des programmes, par courriel à anne-marie.poulin@cihr-irsc.gc.ca ou par téléphone au 613-948-2899.

Nous vous souhaitons un franc succès dans vos travaux de recherche.

Veuillez agréer, Docteure DALLAIRE, l'expression de mes meilleurs sentiments.

Alison Bourgon

Alison Bourgon
Directrice adjointe intérimaire, Direction des programmes de création de connaissances
Portefeuille de la recherche

c.c. Agent financier, Institution payée
Administrateur de recherches, Institution payée

298966-201111PHE-PHE-267464-94456-PHEA

CIHR IRSC

Institute of Aboriginal Peoples' Health
Institute of Aging
Institute of Cancer Research
Institute of Circulatory and Respiratory Health
Institute of Gender and Health
Institute of Genetics
Institute of Health Services and Policy Research
Institute of Human Development and Child and Youth Health
Institute of Infection and Immunity
Institute of Musculoskeletal Health and Arthritis
Institute of Neurosciences, Mental Health and Addiction
Institute of Nutrition, Metabolism and Diabetes
Institute of Population and Public Health

Institut de la santé des Autochtones
Institut du vieillissement
Institut du cancer
Institut de la santé circulatoire et respiratoire
Institut de la santé des femmes et des hommes
Institut de génétique
Institut des services et des politiques de la santé
Institut du développement et de la santé des enfants et des adolescents
Institut des maladies infectieuses et immunitaires
Institut de l'appareil locomoteur et de l'arthrite
Institut des neurosciences, de la santé mentale et des toxicomanies
Institut de la nutrition, du métabolisme et du diabète
Institut de la santé publique et des populations

Le 30 avril 2012

Docteure Clémence DALLAIRE
Faculté des sciences infirmières
1050, rue de la Médecine
Pavillon Ferdinand Vandry
Université Laval
Québec, Québec G1V 0A6

Docteure DALLAIRE,

Nous vous félicitons de votre succès au dernier concours de financement des Instituts de recherche en santé du Canada. Vous devez en être fier, compte tenu de la nature très compétitive de l'évaluation par les pairs des IRSC.

Comme vous le savez, l'évaluation par les pairs est la pierre angulaire de notre système de financement de la recherche. Ce processus repose sur la bonne volonté de vos collègues d'autres établissements, qui ont donné généreusement de leur temps pour évaluer votre demande.

Les Instituts de recherche en santé du Canada ont pour mandat de mettre en oeuvre une entreprise nationale de recherche en santé novatrice. À cette fin, nous avons entrepris l'élaboration d'un plan stratégique renouvelé pour les IRSC, qui a nécessité l'appui des chercheurs, des décideurs, du secteur bénévole et du public. Le partage des connaissances est essentiel pour atteindre l'objectif des IRSC. C'est pourquoi nous vous encourageons à collaborer avec votre établissement afin d'informer la population canadienne des résultats de vos travaux de recherche. Pour simplifier le processus, nous avons élaboré des lignes directrices sur les communications publiques que vous trouverez dans notre site Web à l'adresse http://www.cihr-irsc.gc.ca/f/30789.html.

Nous vous félicitons une fois de plus et vous souhaitons la meilleure des chances dans vos travaux de recherche.

Veuillez agréer, Docteure DALLAIRE, nos salutations distinguées.

Alain Beaudet, M.D., Ph.D.
Président

298968-201111PHE-PHE-267464-94456-CONGR

President

Canadian Institutes of Health Research
Room 97, 160 Elgin Steet, Address locator: 4809A
Ottawa, (Ontario) K1A 0W9 Tel.: (613) 941-2672
Fax (613) 954-1800 **www.cihr-irsc.gc.ca**

Président

Instituts de recherche en santé du Canada
Pièce 97, 160 rue Elgin, Indice de l'adresse: 4809A
Ottawa, (Ontario) K1A 0W9 Tél.: (613) 941-2672
Fax (613) 954-1800 **www.irsc-cihr.gc.ca**

Canada

Application Number / Numéro de demande: 267464
Name of Applicant / Nom du chercheur: DALLAIRE, Clémence
Review Type / Type dévaluation: Committee Member 1/Membre de comité 1
Competition: 2011-11-01 Partnerships for Health System Improvement (PHSI)
Concours: 2011-11-01 Partenariats pour l'amélioration du système de santé (PASS)
Committee: Partnerships for Health System Improvement
Comité: Partenariats pour l'amélioration du système de santé

Potential Impact

Comments:

Le projet vise à évaluer l'implantation d'une clinique de suivis intégrés de maladies chroniques en soins secondaires. Les objectifs spécifiques de la recherche sont : 1) De décrire et d’analyser les modes actuels de pratiques et les arrangements aptes à être pris dans les activités sous-jacentes aux 4 composantes du CCM pour chacune des maladies étudiées. 2) De comprendre les pratiques organisationnelles et cliniques qui en résultent en cours d’implantation de chacune des maladies étudiées. 3) D’évaluer les résultats sur les patients pour chacune des maladies étudiées. 4) De comparer et contraster les pratiques organisationnelles et cliniques de chacune des maladies pour en dégager ce qui en résulte pour les deux maladies étudiées. 5) D’élaborer un modèle de réorganisation de l’offre de services aux personnes atteintes de maladies chroniques. Pour atteindre ces objectifs, une étude de cas comparative longitudinale sera effectuée en utilisant une stratégie mixte de recherche, qualitative et quantitative. Les sources de données pour les objectifs 1 et 2 seront les documents de gestion, des entrevues semi-dirigées (initialement et 6, 12 et 18 mois plus tard) et l’observation de comités pertinents. Pour évaluer les résultats sur les patients, les dossiers patients et des questionnaires seront administrés (outils cliniques et questionnaires sur la satisfaction de l’enseignement à l’autogestion), à 12 et 18 mois après l’implantation.

L’amélioration de la prise en charge des maladies chroniques de façon plus intégrée et efficace est importante pour notre système de santé car au Canada les maladies chroniques coûtent chers et les accès à des services de qualité sont difficiles. L’implantation d’offre de service intégrée et de trajectoires de soins efficaces (respectant le Modèle de soin des maladies chroniques) devrait permettre d’améliorer la qualité des soins et de sauver des coûts.

Le fait que ce soit les décideurs qui ont demandé à l’équipe de recherche de mener cette étude traduit l’importance qu’ils accordent aux conclusions et la forte probabilité qu’ils utilisent ces conclusions. L’équipe de recherche a déjà collaboré avec les décideurs par le passé et un travail préliminaire a été effectué par l’équipe de recherche en collaboration avec les décideurs suite à une Subvention pour réunions, planification et dissémination (SRPD-PASS) des IRSC en avril 2011. Les décideurs sont aussi impliqués tout au long du projet en participant au comité de coordination.

L’impact du projet sur le système de santé sera limité au CHA de Québec et il ne sera pas possible de généraliser la description de l’implantation à d’autres situations. Le projet vise à élaborer un modèle théorique de réorganisation de l’offre de services aux personnes atteintes de maladies chroniques, qui pourrait être généralisable, mais ce modèle nécessitera une validation dans d’autres organisations du système de santé.

Application Number / Numéro de demande: 267464
Name of Applicant / Nom du chercheur: DALLAIRE, Clémence
Review Type / Type dévaluation: Committee Member 1/Membre de comité 1
Competition: 2011-11-01 Partnerships for Health System Improvement (PHSI)
Concours: 2011-11-01 Partenariats pour l'amélioration du système de santé (PASS)
Committee: Partnerships for Health System Improvement
Comité: Partenariats pour l'amélioration du système de santé

Scientific Merit

Comments:

La question de recherche répond clairement aux objectifs de la possibilité de financement et a été identifiée par les décideurs comme étant pertinente.

Le design mixte de l'étude, soit quantitatif pour l'objectif #3 mais par ailleurs essentiellement qualitatif, est approprié pour atteindre les objectifs proposés et les méthodologies sont bien adaptées, variées et bien pensées. Le plan de transfert des connaissances (TC) est bien détaillé, vaste et complet. L'équipe de recherche a une bonne expérience des activités de TC.

Pour les objectifs #1 et 2, les données de gestion et d'entrevue devraient être comparées à des normes établies ou aux données obtenues dans des cliniques similaires dans d'autres centres. I.e. étendre l'étude de 2 cas à d'autres centres. Cela permettrait de déterminer si les modes de pratiques observés sont conformes aux normes (évaluation normative) ou à ce que l'on observe dans une autre clinique similaire qui performe bien, ou encore sont plus performants que dans une clinique usuelle sans suivi intégré des maladies chroniques.

La méthodologie de recherche quantitative qui sera utilisée pour atteindre l'objectif #3 est toutefois peu précise. Il n'est pas fait mention du nombre de patients qu'il est prévu de recruter et si ce nombre sera suffisant pour avoir la précision nécessaire pour que les résultats aient un impact. Comme il n'y a pas de groupe contrôle, il sera difficile de déterminer si les résultats des patients ont une signification quelconque, à moins de se référer à des normes ou des résultats historiques, ce qui n'est pas décrit. Les données auraient aussi pu être comparées avec les résultats dans d'autres milieux, tel que mentionné précédemment, comme groupes contrôles externes. Étant donné le fardeau économique important des maladies chroniques sur le système de santé, une évaluation coût-bénéfice aurait était intéressante.

Le dossier de publication et de subvention de l'équipe est faible. Il n'est pas clair que les chercheurs pourront adéquatement généraliser leurs résultats et les diffuser dans des journaux ayant un impact significatif sur la communauté scientifique. L'équipe regroupe plusieurs expertises importantes, notamment en évaluation qualitative et en gestion/organisation des systèmes de santé. Je note toutefois qu'aucun expert ou clinicien dans le domaine de la diabétologie ne fait parti de l'équipe, alors que le diabète est une des 2 maladies chroniques ciblées. Quant à l'autre maladie chronique, un gastro-entérologue semble faire parti de l'équipe, mais son nom n'est pas mentionné et il n'est pas co-chercheur du projet.

Le projet tel que défini est réalisable avec les ressources et dans les délais proposés.

Le budget est approprié.

Application Number / Numéro de demande: 267464
Name of Applicant / Nom du chercheur: DALLAIRE, Clémence
Review Type / Type dévaluation: Committee Member 2/Membre de comité 2
Competition: 2011-11-01 Partnerships for Health System Improvement (PHSI)
Concours: 2011-11-01 Partenariats pour l'amélioration du système de santé (PASS)
Committee: Partnerships for Health System Improvement
Comité: Partenariats pour l'amélioration du système de santé

Potential Impact

Comments:

The development of integrated care models for chronic diseases is a high priority for healthcare systems world-wide. The timeframe of 3 years is appropriate – somewhat disappointing that the project will end with the design of a model vs. pilot and evaluation of a new model, but the evidence and experience elsewhere indicates that more work up front would ensure effectiveness and sustainability for a new model designed when armed with this information... this end point will likely lead to ‘need more research’; perhaps if successful, an extension of this study would be a good candidate for renewal for a subsequent phase to implement and evaluate this model.

This project builds on a good base of existing partnerships between the U of Laval and local healthcare institutions. This team had received a planning grant to develop the initial project idea, ascertain the current context, and establish partnerships. The decision makers involved are well positioned to use the knowledge gained from this study and implement health system changes as appropriate. One of the decision makers has experience working as a knowledge broker; the other is the DPS and is responsible for quality improvement – so the decision makers on the team are exactly right.

This work is highly likely to lead to the development of a model incorporating evidence-informed changes at the local level for the 2 selected clinical services – diabetes and IBD. Given the focus on care processes and change management, it is very likely that the knowledge about the design and implementation of the services will be transferrable to other clinical priorities, as well as to other institutions and jurisdictions.

**Application Number / Numéro de demande:**	267464
**Name of Applicant / Nom du chercheur:**	DALLAIRE, Clémence
**Review Type / Type dévaluation:**	Committee Member 2/Membre de comité 2
**Competition:**	2011-11-01 Partnerships for Health System Improvement (PHSI)
**Concours:**	2011-11-01 Partenariats pour l'amélioration du système de santé (PASS)
**Committee:**	Partnerships for Health System Improvement
**Comité:**	Partenariats pour l'amélioration du système de santé

Scientific Merit

Comments:

This project focuses on harmonizing interventions for 2 chronic diseases at the 2 facilities that make up the CHA, and on 4 aspects that have been shown to be linked to success: the organization of service delivery (i.e. provider roles, interdisciplinary teams, and coordination of follow-up), support for self-care, support for clinical decision-making, and the development of clinical information systems.

Overall objectives – to obtain new knowledge from the study of 2 chronic diseases, to enable the institutions to implement an integrated chronic care model more broadly; to develop an explanatory theory that would apply to other jurisdictions or 'cases' and inform the development of programs such as this elsewhere.

Team proposes primarily a case study approach using mixed methods; appropriate for this study.
Patient surveys are using standardised tools; didn't see sample size(s) proposed??.
In summary, it is a relatively long, baseline measurement study that attempts to obtain a deep understanding of the care processes, context, and professional interactions/behaviours that affect patient care, to inform the design of a new model.

The research lead has excellent background in applied research and KT and has the appropriate expertise and experience to lead the scientific and partnership aspects of this project. The team is multidisciplinary with appropriate range of disciplines and skills for this project, and includes a doctoral student who will benefit from participation on this team. Interestingly, there is a Brazilian researcher as a member of the team who will conduct some comparative analysis which will be helpful to the team and add another dimension to the reporting of results, but not really clear what role is on the team. The team has included a GI specialist who will provide advice and research support on the clinical aspects; he has a particular interest in outcomes measurement and he obtained funding to hire an RN for the team.

KT – via advisory committee throughout project; development of workshop near end of project – to apply lessons and develop service model. Conference attendance requested every year.

Total funding request: $304,643 ($97,932 matched funds (32%) – in-kind)
Items and amounts appear reasonable over the life of the award.

**Review Type/Type dévaluation:**	SO Notes /Notes de l'agent scientifique
**Name of Applicant/Nom du chercheur:**	DALLAIRE, Clémence
**Application No./Numéro de demande:**	267464
**Agency/Agence:**	CIHR/IRSC
**Competition/Concours:**	2011-11-01 Partnerships for Health System Improvement (PHSI)/Partenariats pour l'amélioration du système de santé (PASS)
**Committee/Comité:**	Partnerships for Health System Improvement/Partenariats pour l'amélioration du système de santé
**Title/Titre:**	L'implantation d'une clinique de suivis intégrés de maladies chroniques en misant sur la deuxieme ligne de soins

Assessment/Évaluation:

The project looks at an integrated chronic disease program using a mixed-methods longitudinal case study design. The policy makers' request to develop the research program and the development through a MPD grant is a strength. The research plan will develop an explanatory theory and general model which will enhance the generalizability of the research in spite of only one program being examined. The study would be strengthened with improved comparison to usual care. The grant and publication record of the team is not strong. The team could also be strengthened with relevant clinician involvement.
