# Supplementary material for: Secondary Care Clinic for Chronic Disease: Protocol
Source: JMIR Res Protoc. 2015 Feb 16;4(1):e12. doi: 10.2196/resprot.3902 (PMC4376234; doi:10.2196/resprot.3902)
Supplement: Supplementary file 3 [file resprot_v4i1e12_app3.pdf]

Application Number / Numéro de demande: 267464
Name of Applicant / Nom du chercheur: DALLAIRE, Clémence
Review Type / Type dévaluation: Committee Member 1/Membre de comité 1
Competition: 2011-11-01 Partnerships for Health System Improvement (PHSI)
Concours: 2011-11-01 Partenariats pour l'amélioration du système de santé (PASS)
Committee: Partnerships for Health System Improvement
Comité: Partenariats pour l'amélioration du système de santé

Potential Impact

Comments:

Le projet vise à évaluer l'implantation d'une clinique de suivis intégrés de maladies chroniques en soins secondaires. Les objectifs spécifiques de la recherche sont : 1) De décrire et d'analyser les modes actuels de pratiques et les arrangements aptes à être pris dans les activités sous-jacentes aux 4 composantes du CCM pour chacune des maladies étudiées. 2) De comprendre les pratiques organisationnelles et cliniques qui en résultent en cours d'implantation de chacune des maladies étudiées. 3) D'évaluer les résultats sur les patients pour chacune des maladies étudiées. 4) De comparer et contraster les pratiques organisationnelles et cliniques de chacune des maladies pour en dégager ce qui en résulte pour les deux maladies étudiées. 5) D'élaborer un modèle de réorganisation de l'offre de services aux personnes atteintes de maladies chroniques. Pour atteindre ces objectifs, une étude de cas comparative longitudinale sera effectuée en utilisant une stratégie mixte de recherche, qualitative et quantitative. Les sources de données pour les objectifs 1 et 2 seront les documents de gestion, des entrevues semi-dirigées (initialement et 6, 12 et 18 mois plus tard) et l'observation de comités pertinents. Pour évaluer les résultats sur les patients, les dossiers patients et des questionnaires seront administrés (outils cliniques et questionnaires sur la satisfaction de l'enseignement à l'autogestion), à 12 et 18 mois après l'implantation.

L'amélioration de la prise en charge des maladies chroniques de façon plus intégrée et efficace est importante pour notre système de santé car au Canada les maladies chroniques coûtent chers et les accès à des services de qualité sont difficiles. L'implantation d'offre de service intégrée et de trajectoires de soins efficaces (respectant le Modèle de soin des maladies chroniques) devrait permettre d'améliorer la qualité des soins et de sauver des coûts.

Le fait que ce soit les décideurs qui ont demandé à l'équipe de recherche de mener cette étude traduit l'importance qu'ils accordent aux conclusions et la forte probabilité qu'ils utilisent ces conclusions. L'équipe de recherche a déjà collaboré avec les décideurs par le passé et un travail préliminaire a été effectué par l'équipe de recherche en collaboration avec les décideurs suite à une Subvention pour réunions, planification et dissémination (SRPD-PASS) des IRSC en avril 2011. Les décideurs sont aussi impliqués tout au long du projet en participant au comité de coordination.

L'impact du projet sur le système de santé sera limité au CHA de Québec et il ne sera pas possible de généraliser la description de l'implantation à d'autres situations. Le projet vise à élaborer un modèle théorique de réorganisation de l'offre de services aux personnes atteintes de maladies chroniques, qui pourrait être généralisable, mais ce modèle nécessitera une validation dans d'autres organisations du système de santé.

**Application Number / Numéro de demande:**	267464
**Name of Applicant / Nom du chercheur:**	DALLAIRE, Clémence
**Review Type / Type dévaluation:**	Committee Member 1/Membre de comité 1
**Competition:**	2011-11-01 Partnerships for Health System Improvement (PHSI)
**Concours:**	2011-11-01 Partenariats pour l'amélioration du système de santé (PASS)
**Committee:**	Partnerships for Health System Improvement
**Comité:**	Partenariats pour l'amélioration du système de santé

Scientific Merit

Comments:

La question de recherche répond clairement aux objectifs de la possibilité de financement et a été identifiée par les décideurs comme étant pertinente.

Le design mixte de l'étude, soit quantitatif pour l'objectif #3 mais par ailleurs essentiellement qualitatif, est approprié pour atteindre les objectifs proposés et les méthodologies sont bien adaptées, variées et bien pensées. Le plan de transfert des connaissances (TC) est bien détaillé, vaste et complet. L'équipe de recherche a une bonne expérience des activités de TC.

Pour les objectifs #1 et 2, les données de gestion et d'entrevue devraient être comparées à des normes établies ou aux données obtenues dans des cliniques similaires dans d'autres centres. I.e. étendre l'étude de 2 cas à d'autres centres. Cela permettrait de déterminer si les modes de pratiques observés sont conformes aux normes (évaluation normative) ou à ce que l'on observe dans une autre clinique similaire qui performe bien, ou encore sont plus performants que dans une clinique usuelle sans suivi intégré des maladies chroniques.

La méthodologie de recherche quantitative qui sera utilisée pour atteindre l'objectif #3 est toutefois peu précise. Il n'est pas fait mention du nombre de patients qu'il est prévu de recruter et si ce nombre sera suffisant pour avoir la précision nécessaire pour que les résultats aient un impact. Comme il n'y a pas de groupe contrôle, il sera difficile de déterminer si les résultats des patients ont une signification quelconque, à moins de se référer à des normes ou des résultats historiques, ce qui n'est pas décrit. Les données auraient aussi pu être comparées avec les résultats dans d'autres milieux, tel que mentionné précédemment, comme groupes contrôles externes. Étant donné le fardeau économique important des maladies chroniques sur le système de santé, une évaluation coût-bénéfice aurait était intéressante.

Le dossier de publication et de subvention de l'équipe est faible. Il n'est pas clair que les chercheurs pourront adéquatement généraliser leurs résultats et les diffuser dans des journaux ayant un impact significatif sur la communauté scientifique. L'équipe regroupe plusieurs expertises importantes, notamment en évaluation qualitative et en gestion/organisation des systèmes de santé. Je note toutefois qu'aucun expert ou clinicien dans le domaine de la diabétologie ne fait parti de l'équipe, alors que le diabète est une des 2 maladies chroniques ciblées. Quant à l'autre maladie chronique, un gastro-entérologue semble faire parti de l'équipe, mais son nom n'est pas mentionné et il n'est pas co-chercheur du projet.

Le projet tel que défini est réalisable avec les ressources et dans les délais proposés.

Le budget est approprié.
