# Supplementary material for: Secondary Care Clinic for Chronic Disease: Protocol
Source: JMIR Res Protoc. 2015 Feb 16;4(1):e12. doi: 10.2196/resprot.3902 (PMC4376234; doi:10.2196/resprot.3902)
Supplement: Supplementary file 4 [file resprot_v4i1e12_app4.pdf]

Application Number / Numéro de demande: 267464
Name of Applicant / Nom du chercheur: DALLAIRE, Clémence
Review Type / Type dévaluation: Committee Member 2/Membre de comité 2
Competition: 2011-11-01 Partnerships for Health System Improvement (PHSI)
Concours: 2011-11-01 Partenariats pour l'amélioration du système de santé (PASS)
Committee: Partnerships for Health System Improvement
Comité: Partenariats pour l'amélioration du système de santé

Potential Impact

Comments:

The development of integrated care models for chronic diseases is a high priority for healthcare systems world-wide. The timeframe of 3 years is appropriate – somewhat disappointing that the project will end with the design of a model vs. pilot and evaluation of a new model, but the evidence and experience elsewhere indicates that more work up front would ensure effectiveness and sustainability for a new model designed when armed with this information... this end point will likely lead to 'need more research'; perhaps if successful, an extension of this study would be a good candidate for renewal for a subsequent phase to implement and evaluate this model.

This project builds on a good base of existing partnerships between the U of Laval and local healthcare institutions. This team had received a planning grant to develop the initial project idea, ascertain the current context, and establish partnerships. The decision makers involved are well positioned to use the knowledge gained from this study and implement health system changes as appropriate. One of the decision makers has experience working as a knowledge broker; the other is the DPS and is responsible for quality improvement – so the decision makers on the team are exactly right.

This work is highly likely to lead to the development of a model incorporating evidence-informed changes at the local level for the 2 selected clinical services – diabetes and IBD. Given the focus on care processes and change management, it is very likely that the knowledge about the design and implementation of the services will be transferrable to other clinical priorities, as well as to other institutions and jurisdictions.

**Application Number / Numéro de demande:**	267464
**Name of Applicant / Nom du chercheur:**	DALLAIRE, Clémence
**Review Type / Type dévaluation:**	Committee Member 2/Membre de comité 2
**Competition:**	2011-11-01 Partnerships for Health System Improvement (PHSI)
**Concours:**	2011-11-01 Partenariats pour l'amélioration du système de santé (PASS)
**Committee:**	Partnerships for Health System Improvement
**Comité:**	Partenariats pour l'amélioration du système de santé

Scientific Merit

Comments:

This project focuses on harmonizing interventions for 2 chronic diseases at the 2 facilities that make up the CHA, and on 4 aspects that have been shown to be linked to success: the organization of service delivery (i.e. provider roles, interdisciplinary teams, and coordination of follow-up), support for self-care, support for clinical decision-making, and the development of clinical information systems.

Overall objectives – to obtain new knowledge from the study of 2 chronic diseases, to enable the institutions to implement an integrated chronic care model more broadly; to develop an explanatory theory that would apply to other jurisdictions or 'cases' and inform the development of programs such as this elsewhere.

Team proposes primarily a case study approach using mixed methods; appropriate for this study.
Patient surveys are using standardised tools; didn't see sample size(s) proposed??.
In summary, it is a relatively long, baseline measurement study that attempts to obtain a deep understanding of the care processes, context, and professional interactions/behaviours that affect patient care, to inform the design of a new model.

The research lead has excellent background in applied research and KT and has the appropriate expertise and experience to lead the scientific and partnership aspects of this project. The team is multidisciplinary with appropriate range of disciplines and skills for this project, and includes a doctoral student who will benefit from participation on this team. Interestingly, there is a Brazilian researcher as a member of the team who will conduct some comparative analysis which will be helpful to the team and add another dimension to the reporting of results, but not really clear what role is on the team. The team has included a GI specialist who will provide advice and research support on the clinical aspects; he has a particular interest in outcomes measurement and he obtained funding to hire an RN for the team.

KT – via advisory committee throughout project; development of workshop near end of project – to apply lessons and develop service model. Conference attendance requested every year.

Total funding request: $304,643 ($97,932 matched funds (32%) – in-kind)
Items and amounts appear reasonable over the life of the award.
